# Markers of subtypes in inflammatory breast cancer studied by immunohistochemistry: Prominent expression of P-cadherin

**DOI:** 10.1186/1471-2407-8-28

**Published:** 2008-01-29

**Authors:** Azza Ben Hamida, Intidhar S Labidi, Karima Mrad, Emmanuelle Charafe-Jauffret, Saïda Ben Arab, Benjamin Esterni, Luc Xerri, Patrice Viens, François Bertucci, Daniel Birnbaum, Jocelyne Jacquemier

**Affiliations:** 1 Centre de Recherche en Cancérologie de Marseille, Département d'Oncologie Moléculaire, UMR599 Inserm; Institut Paoli-Calmettes; IFR137, Marseille, France; 2 Département de Médecine, Institut Salah Azaiz, Tunis, Tunisie; 3 Unité d'Epidémiologie Génétique et Moléculaire, Faculté de Médecine, Tunis, Tunisie; 4 Département de Pathologie, Institut Salah Azaiz, Tunis, Tunisie; 5 Département de BioPathologie, Institut Paoli-Calmettes, Marseille, France; 6 UFR de Médecine, Université de la Méditerranée, Marseille, France; 7 Département de Biostatistiques, Institut Paoli-Calmettes, Marseille, France; 8 Département d'Oncologie Médicale, Institut Paoli-Calmettes, Marseille, France

## Abstract

**Background:**

Inflammatory breast cancer (IBC) is a distinct and aggressive form of locally-advanced breast cancer with high metastatic potential. In Tunisia, IBC is associated with a high death rate. Among the major molecular subtypes, basal breast carcinomas are poorly differentiated, have metastatic potential and poor prognosis, but respond relatively well to chemotherapy. The aim of this study was to determine the distribution of molecular subtypes in IBC and identify factors that may explain the poor prognosis of IBC.

**Methods:**

To determine breast cancer subtypes we studied by immunohistochemistry the expression of 12 proteins in a series of 91 Tunisian IBC and 541 non-IBC deposited in tissue microarrays.

**Results:**

We considered infiltrating ductal cases only. We found 33.8% of basal cases in IBC vs 15.9% in non-IBC (p < 0.001), 33.3% of ERBB2-overexpressing cases in IBC vs 14.5% in non-IBC (p < 0.001), and 29.3% of luminal cases in IBC vs 59.9% in non-IBC (p < 0.001). The most differentially-expressed protein between IBCs and non-IBCs was P-cadherin. P-cadherin expression was found in 75.9% of all IBC vs 48.2% of all non-IBC (p < 0.001), 95% of IBC vs 69% of non-IBC (p = 0.02) in basal cases, and 82% of IBC vs 43% of non-IBC (p < 0.001) in luminal cases. Logistic regression determined that the most discriminating markers between IBCs and non-IBCs were P-cadherin (OR = 4.9, p = 0.0019) MIB1 (OR = 3.6, p = 0.001), CK14 (OR = 2.7, p = 0.02), and ERBB2 (OR = 2.3, p = 0.06).

**Conclusion:**

Tunisian IBCs are characterized by frequent basal and ERBB2 phenotypes. Surprisingly, luminal IBC also express the basal marker P-cadherin. This profile suggests a specificity that needs further investigation.

## Background

Inflammatory breast cancer (IBC) represents less than 10% of all breast cancers but is the most lethal form of the disease [1]. Evolution of this distinct and aggressive form of locally-advanced breast cancer depends on the type of neo-adjuvant chemotherapy but is overall poor [2]. IBC, classified T4d (stage IIIB) according to the TNM classification of the American Joint Committee of Cancer [5], is generally a rapidly growing tumor associated with cutaneous erythema and edema [2-4]. The inflammatory aspect is associated with highly angiogenic and angio-invasive properties and with the presence of dermal lymphatic emboli [5]. The high metastatic potential of IBCs suggests a high propensity of the tumor cells to migrate.

IBCs in Tunisia are characterized by a higher frequency and a higher aggressiveness than in European countries [6-8]. However, we did not observe any difference between Tunisian and French IBCs in the expression of five representative proteins (E-cadherin, Estrogen receptor (ER), MIB1, MUC1 and ERBB2) [9,10].

In non-IBCs the five molecular subtypes (luminal A and B, basal, ERBB2-overexpressing and normal-like) are associated with different features, including response to chemotherapy and clinical outcome [11]. Basal and ERBB2 subtypes have the worst prognosis, followed by luminal B subtype. The proportion of these subtypes has been determined by gene expression profiling in European IBC. Around half the cases are of basal and ERBB2 subtypes.

The subtypes defined by gene expression analysis have also been defined at the protein level using various markers. A panel of antibodies (directed against ER, ERBB2, EGFR, basal cytokeratin CK5/6 and/or KIT) identifies basal tumors with high specifity [12]. Other markers such as P-cadherin, CK14, P53, CAV1, PR, MIB1 and moesin also identify basal tumors [13-16]. Reciprocally, the expression of ER and ERBB2 exclude basal tumors.

The high frequency of the two subtypes associated with poor prognosis, basal and ERBB2, explains only in part the fatal evolution of IBC. In IBCs, the luminal cases also have a poor prognosis. Only a factor common to all subtypes could explain IBC poor evolution.

In breast cancer, P-cadherin is associated with enhanced cell invasion, tumor aggressiveness, motility [17] and with a poor prognosis [18,19]. P-cadherin mRNA is essentially expressed in basal carcinomas [20,21].

The aims of this study were to determine the importance of breast carcinoma subtypes in IBC and to identify a factor that could explain the poor prognosis of IBC. We show that this factor could be associated with P-cadherin expression.

## Methods

### Definition and selection of cases

We collected a series of 91 Tunisian T4d tumors (TNM, UICC) treated between 1994 and 1998 at the Salah Azaiz Institute (ISA, Tunis, Tunisia). Paraffin-embedded specimens were collected prior chemotherapy. The presence of dermal lymphatic emboli was not mandatory. All patients received neo-adjuvant chemotherapy with anthracyclin-based (FEC 100 or FAC 50) regimen. Forty-three patients were metastatic at diagnosis and did not have complementary mastectomy. External beam radiation was done for all patients.

This series was compared with a consecutive series of 547 non-IBC cases deposited in tissue microarrays and previously used in a study of IBC cases that established IBC immunohistochemical profile [22] and for other purposes [23]. The 547 non-IBC cases were selected from cases included in the database of the Paoli-Calmettes Institute (IPC, Marseille, France) treated between 1990 and 1999. They were defined as T1, T2, T3 tumors (TNM, UICC), collected prior to adjuvant chemotherapy and embedded in paraffin.

### Clinicopathological study

The clinical records were reviewed to determine the following patient characteristics: age, metastatic status, therapeutic regimen and survival. Pathological slides were reviewed by two pathologists (JJ, KM) according to the European guidelines [24]. Criteria evaluated were histological type, Elston-and-Ellis grade and, whenever possible, peritumoral vascular invasion.

### Tissue Microarray construction

Two tissue microarrays (TMA) were used, one for the IBCs (ISA set) and the other one for the non-IBCs (IPC set). TMAs were prepared as described, with slight modifications [22-25]. Briefly, for each tumor, three representative tumor areas were carefully selected from a hematoxylin-eosin-safran stained section of a donor block. Core cylinders with a diameter of 0.6 mm each were punched from each of these areas and deposited into a recipient paraffin block using a specific arraying device (Alphelys, Plaisir, France). In addition to tumor tissues, the recipient blocks also received normal breast tissues and cell lines. Five-μm sections of the resulting microarray blocks were made and used for IHC analysis after transfer to glass slides.

### Markers and immunohistochemistry

Immunohistochemistry (IHC) was performed on 5-μm sections. The characteristics of the antibodies used and pre-treatment conditions are listed in Table 1. A good concordance of IHC results has been reported between standard full tissue sections and TMA [21]. TMA data were evaluated by the mean score of two cores biopsies minimum for each case. The slides were dewaxed, pre-treated according to the supplier's recommendations (Table 1). This was followed by the use of a streptavidin/biotin kit (Dako, Trappes, France). Diaminobenzidine, (DAB) or 3-amino-9-ethylcarbazole (AEC) was used as chromogen. Sections were counterstained with hematoxylin and coverslipped. Slides were evaluated under a light microscope by two independent observers on the Spot Browser device (Alphelys). Immunoreactivities were classified by estimating the percentage (P) of tumor cells showing characteristic staining (from 0%, undetectable level, to 100%, homogeneous staining) and by estimating the intensity (I) of staining 1, weak staining; 2, moderate staining; 3, strong staining). Results were scored by multiplying the percentage of positive cells by the intensity, i.e. by the so-called quick-score (Q). Internal positive controls such as epidermis or benign breast lobules were used. Hormone receptors (ER, PR) were positive when at least 1% of tumor cell nuclei were stained. For ERBB2, the Dako scale was used; staining was considered as positive when limited to a membrane staining of more than 10% of tumor cells and scored as 1+, 2+ or 3+ according to intensity and partial/complete staining. Protein overexpression was considered for 2+ and 3+. Cytokeratin 5/6, cytokeratin 14 and EGFR were scored positive if any (weak or strong) membranous invasive carcinoma cell staining was observed. Caveolin 1 (CAV1) and Caveolin 2 (CAV2) status of tumor cells was evaluated by light microscopy as either positive (any tumor cell with IHC staining) or negative. For CAV1 negative cases, endothelial cells and interstitial fibroblasts were used as internal positive controls.

**Table 1:** Main characteristics of the antibodies used in immunohistochemistry.

Protein (Clone)	Antibody	Origin	Clone	Pre-treatment	Dilution	Location of staining	Normal
Caveolin 1	mmb	Santa Cruz	N20	Micro waves (10 min), tps citrate pH = 6	1/1000	cytoplasm	m.e.c+
Caveolin 2	mmb	Transduction Laboratories	65	Target retrival solution (98°C, 40 min)	1/50	cytoplasm	m.e.c+
Cytokeratin 5/6	mmb	Dako	D5/16 B4	Target retrival solution (98°C, 40 min)	1/10	cytoplasm	m.e.c +
Cytokeratin 14	mmb	Newcastle UK	LL002	Target retrival solution (98°C, 40 min)	1/300	Cell membrane	m.e.c+
EGFR	mmb	Zymed	3IG7	Target retrival solution (98°C, 40 min)	1/10	membrane	m.e.c +
ERBB2	mmb	Dako Herceptest Ltd	AO485	Target retrival solution (98°C, 40 min)	1/500	membrane	-
Estrogen receptor (ER)	mmb	Novocastra laboratories Ltd	6F11.2	Target retrival solution (98°C, 40 min)	1/60	nucleus	m.e.c- and luminal+
P53	mmb	Dako	DO-1	Target retrival solution (98°C, 40 min)	1/4	nucleus	-
P-cadherin	mmb	Transduction laboratories	56	Target retrival solution (98°C, 40 min)	1/75	Cell membrane	m.e.c +
Progesterone receptor (PR)	mmb	Dako	PFR 636	Target retrival solution (98°C, 40 min)	1/80	Nucleus	m.e.c- and luminal+
Ki67	mmb	Dako	KI-67	Target retrival solution (98°C, 40 min)	1/100	Nuclear	-
MUC1	mmb	Transgen	H23	none	1/1000	Apical/Cytoplamic	+

mmb: mouse monoclonal antibody

m.e.c: myoepithelials cells

### Statistical analysis

Data were summarized by frequencies and percentages for categorical variables. Furthermore, for continuous variables the means, the median and range were computed. To investigate the association between categorical variables, univariate statistical analysis were done, using Pearson's Chi-2 test or Fisher's exact test for small sample size and using non-parametric Mann and Whitney test for continuous variables [26]. Multivariate analysis was done using logistic regression model with backward stepwise selection procedure to evaluate the effect of interactions between the different variables. All statistical tests were two-sided at the 5% level of significance. All the statistical analyses were done using R.2.4.0 statistical software[27].

## Results

### Characteristics of patients and tumors

A total of 91 IBC and 547 non-IBC informative cases from women patients were analyzed. Their characteristics are listed in Table 2. Age was different (p < 0.001) for the two series: for IBCs, age ranged from 22 to 76 years (mean 43.47 years) whereas for non-IBCs, age ranged from 25 to 94 years (mean 59.43 years).

**Table 2:** Clinical and histological characteristics of IBC and non-IBC cases.

Variables	IBC	Non-IBC	p
Age			
Min	22	25	
Max	76	94	<0.001
Mean	43.47	59.43	
Standard deviation	9.43	12.77	
Grade			
I	3(3.3%)	176(32.41%)	
II	60(65.93%)	229(42.17%)	<0.001
III	28(30.77%)	138(25.41%) *	
Histological type			
Invasive ductal carcinoma	86(94.5)	386(70.56)	
Micropapillary carcinoma	5(5.5)	0	
Lobular carcinoma	0	72(13.16)	
Tubular carcinoma	0	37(6.76)	<0.001
Mixt	0	24(4.38)	
Medullary carcinoma	0	8(1.46)	
Others	0	20(3.65)	

* 4 cases non valuable for the histoprognostic grade.

Among the 91 IBCs, 86 were invasive ductal carcinomas (IDC) and 5 were invasive micropapillary carcinomas. The distribution of histological types in non-IBCs was representative of unbiased populations. IBCs were more frequently of grade II and III (65.93%, 30.77%) than non-IBCs (p < 0.001) (Table 2).

### Protein expression profiles of IBC and non-IBC and immunohistochemical subtypes

**Figure 1:**
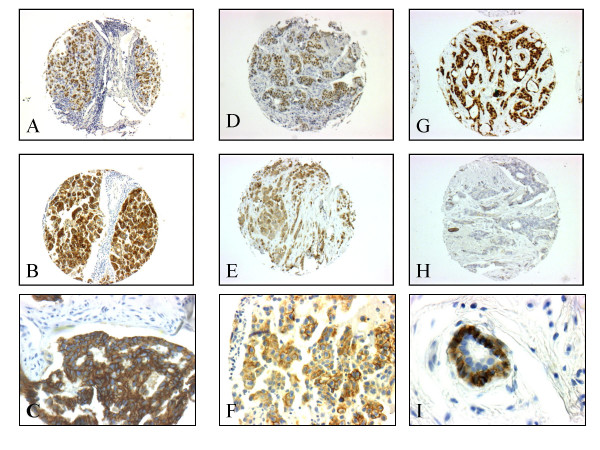
**Immunohistochemistry on tissue microarray sections of breast cancers of the luminal subtype**. A: Expression of estrogen receptor in 100% of tumor cells in a grade III inflammatory case. B: Strong expression of P-cadherin in 100% of tumor cells in the same case. C: Higher magnification showing the cell membrane localization. D: Expression of estrogen receptor in 100 % of tumor cells in a more differentiated (grade II) inflammatory case. E: Moderate expression of P- cadherin in 100% of tumor cells in the same case. F: Higher magnification showing the cell membrane localization. G: Luminal grade II non inflammatory case with 100% estrogen receptor-positive cells. H: Absence of expression of P-cadherin in the same non inflammatory case; note the positive internal control on myoepithelial cells of the normal duct. I: Higher magnification showing the cell membrane localization of the myoepithelial cells by contrast to the negativity of the luminal normal cells.

IHC was done on IBCs and non-IBCs using three TMA slides for each marker. We compared the expression of the markers only for the infiltrating ductal cases, i.e. 86 IBC cases and 386 non-IBC cases. We observed differences between the two series for: ER negativity (53.85% IBCs, 26.26 % non-IBCs; p < 0.001), PR negativity (52.86% IBCs, 35.39% non-IBCs; p < 0.01), ERBB2 positivity (33.33% IBCs, 14.49% non-IBCs; p < 0.001), CK14 positivity (19.15% IBCs, 5.45% non-IBCs; p < 0.01), P-cadherin positivity (75.93% IBCs, 48.16% non-IBCs; p < 0.001) (Figure 1) and proliferative rate expressed by a Ki67 index (MIB1) higher than 20% (41.1% IBCs, 12.98% non-IBCs; p < 0.001) (Table 3).

**Table 3:** Immunohistochemical comparison of the different markers used in the two series

		IBC	Non-IBC	p value
		n (%)	n (%)	
ER	Negative	42 (53.85%)	99 (26.26%)	<0.001
	Positive	36 (46.15%)	278 (73.74%)	
PR	Negative	37 (52.86%)	132 (35.39%)	<0.01
	Positive	33 (47.14%)	241 (64.61%)	
ERBB2	0–1	48 (66.67%)	301 (85.51%)	<0.001
	2–3	24 (33.33%)	51 (14.49%)	
EGFR	Negative	57 (77.03%)	240 (75.24%)	>0.05
	Positive	17 (22.97%)	79 (24.76%)	
CK5/6	Negative	47 (61.04%)	191 (67.25%)	>0.05
	Positive	30 (38.96%)	93 (32.75%)	
CK14	Negative	38 (80.85%)	295 (94.55%)	<0.01
	Positive	9 (19.15%)	17 (5.45%)	
P-cadherin	Negative	13 (24.07%)	169 (51.84%)	<0.001
	Positive	41 (75.93%)	157 (48.16%)	
P53	Negative	43 (58.11%)	253 (69.7%)	>0.05
	Positive	31 (41.89%)	110 (30.3%)	
MUC1	Negative	5 (6.67%)	42 (12.84%)	>0.05
	Positive	70 (93.33%)	285 (87.16%)	
CAV1	Negative	22 (31.43%)	105 (31.16%)	>0.05
	Positive	48 (68.57%)	232 (68.84%)	
CAV2	Negative	45 (90%)	212 (89.83%)	>0.05
	Positive	5 (10%)	35 (10.17%)	
MIB1	≤20	43 (58.9%)	295 (87.02%)	<0.001
	>20	30 (41.1%)	44 (12.98%)	

We classified tumors in terms of subtypes based on ER/PR and ERBB2 IHC expression [12,28]. The analysis was restricted to the infiltrating ductal cases (86 IBC and 386 non-IBC), and allowed a classification in three groups (Table 4). ER-positive and PR-positive tumors were classified as luminal. ERBB2-positive tumors were classified as ERBB2-overexpressing. ER-negative and ERBB2-negative (double negative) were considered as basal (ER-/ERBB2-). Using this protein-based definition of subtypes, 33.8% of IBCs vs 15.93 % of non-IBCs (p < 0.001) were basal, 33.33 % of IBCs vs 14.49% of non-IBCs (p < 0.001) were ERBB2-overexpressing, and 29.33% of IBCs vs 58.93% of non-IBCs (p < 0.001) were luminal.

**Table 4:** Immunohistochemical classification in molecular subtypes of the two series

	IBC	Non-IBC	
		
	n (%)	n (%)	p value
Basal cases (ER-, ERBB2-)	24 (33.8%)	58 (15.93%)	<0.001
ERBB2-overexpressing cases	24 (33.33%)	51 (14.49%)	<0.001
Luminal cases (ER+, PR+)	22 (29.33%)	221 (58.93%)	<0.001

### Expression of P-cadherin in IBC and non-IBC

#### Univariate analyses

Among various markers tested, the expression of P-cadherin was worth further investigation. The expression of P-cadherin varied according to the subtype and the IBC status in infiltrating ductal cases. The expression of P-cadherin in the basal subtype was 95% for IBCs vs 69% for non-IBCs (p = 0.02). There was no difference between IBCs and non-IBCs in the ERBB2-overexpressing subtype. In luminal tumors, P-cadherin was expressed in 82 % of IBCs vs 43% of non-IBCs (p < 0.01) (Table 5). Finally, we found a strong correlation between P-cadherin and Ki67/MIB1 (p = 0.0004).

**Table 5:** Comparison of P-cadherin expression in different breast cancer subtypes

	IBC	Non-IBC	p-value
		
	n(%)	n(%)	
Basal cases (ER-,ERBB2-)	18/19(95%)	35/51(69%)	0.05
ERBB2-overexpressing cases	9/13(69%)	32/47(68%)	NS
Luminal cases (ER+, PR+)	14/17(82%)	84/197(43%)	<0.01

#### Multivariate analyses

We used a logistic regression stepwise selection with clinical and biological factors (Table 6). The most discriminant factors for IBC status were grade (Odd ratio, OR = 6.28, p = 0.081), and P-cadherin (OR = 4.21, p = 0.013), MIB1 (OR = 2.82, p = 0.018), and ER (OR = 1.00, p = 0.053) expression. In a logistic regression by backward stepwise selection using biological factors only, the factors discriminating IBC from non-IBC were P-cadherin (OR = 4.9, p = 0.0019), MIB1 (OR = 3.6, p = 0.001), CK14 (OR = 2.7, p = 0.02), and ERBB2 (OR = 2.3, p = 0.06).

**Table 6:** Logistic regression using prognostic and biological factors to best determine IBC status.

With histoprognostic factors	Regression coefficient	OR	p	Without histoprognostic factors	Regression coefficient	OR	p
Grade (II-III)	1.84	6.28	0.081	P-cadherin >0	1.59	4.90	0.001
P-cadherin >0	1.44	4.21	0.013	MIB 1 >20	1.28	3.60	0.001
MIB1 >20	1.04	2.82	0.018	CK14 >0	1.02	2.79	0.028
ER <0	-0.87	1.00	0.053	ERBB2 (2–3)	0.84	2.34	0.063

## Discussion

The identification of a specific IBC profile could improve the diagnosis, treatment and evolution of this aggressive form of locally-advanced breast cancer with high metastatic potential. In Tunisia, IBCs are frequent and have a worse prognosis than in European countries. However, in a previous study, we did not observe any difference in protein expression between IBCs from France and Tunisia [10]. Five major molecular subtypes have been defined by gene expression profiling [11,20,29]. Basal and ERBB2 subtypes represent each about 20% of cases. These subtypes could also be recognized at the protein level, with a very good correlation with RNA analyses. IHC-defined subtypes also show differences in prognosis in non-IBCs: basal and ERBB2-overexpressing subtypes are associated with poor prognosis. To better understand the specificity and the poor prognostic of IBCs in general and Tunisian IBCs in particular, we studied by IHC on TMA the expression of proteins commonly used as markers for molecular subtypes.

We studied several markers but eventually used a simple operational definition based on ER and ERBB2 expression. The basal subtype was defined as negative for both ER and ERBB2. In non-IBCs basal and ERBB2 subtypes represent around 40% of cases. We compared only infiltrating ductal cases. We found differences in the proportion of subtypes in Tunisian IBCs. Basal and ERBB2 subtypes made up each about one-third of the cases. Overall, these data and those of the literature show an over-representation of basal and ERBB2 cases in IBCs as compared with non-IBCs [30,31].

However, the high proportion of basal and ERBB2 subtypes is not sufficient *per se *to explain IBC prognosis. Only a factor common to all subtypes could explain IBC poor evolution. Surprisingly, we found that luminal IBCs express basal markers such as P-cadherin. Indeed, P-cadherin expression was not only higher in basal IBCs than in basal non-IBCs (95%/69%) but also higher in the IBC luminal cases (82%/42%). The logistic regression analysis showed that P-cadherin was the most representative marker of IBC.

P-cadherin is one of the most specific markers of myoepithelial cells and is associated with basal subtype. The frequency of P-cadherin IHC expression varies from 20% in initial works [32] to 40% in more recent series[33]. This percentage exceeds that of basal tumors suggesting that P-cadherin could also be expressed in non-basal cases. P-cadherin expression in breast cancer correlates with high grade, lack of ER/PR expression, increased tumor aggressiveness, high proliferation rate, and poor survival. P-cadherin is associated with MIB1, EGFR, ERBB2, P53 and CK5/6 expression [23]. This expression may be due to hypomethylation of the gene promoter [18]. Deregulated P-cadherin expression may alter epithelial cell behavior thereby contributing to a more aggressive tumor cell phenotype and poor survival [34]. P-cadherin has pro-invasive activity in the MCF-7/AZ luminal breast cancer cell line, through interaction with signaling proteins bound to its juxtamembrane domain. ICI182,780, which blocks ER, induces increased expression of P-cadherin, which is associated with *in vitro *invasion[35]. Overexpressed P-cadherin increases motility of pancreatic cancer cells by interacting with p120ctn and subsequent activation of RHO GTPases [36]. Activation of RHO GTPases has been observed in IBC. Overexpression of RHOC is associated with the loss of expression of WISP3 [37,38], which acts as a tumor suppressor. RHOC is overexpressed in over 90% of IBCs vs 36% of non-IBCs. RHOC activity requires NFκB stimulation [39]. P-cadherin transgenic mice do not develop mammary tumors spontaneously. When mammary tumors are induced in the P-cadherin transgenic mice through breeding with the MMTV/neu transgenic mouse, the tumors do not express P-cadherin. This indicates that P-cadherin is not *per se *an oncogene [34].

## Conclusion

Expression of P-cadherin in luminal IBC may indicate that the tumor cells derive from a stem/basal cell that has acquired ER expression and a luminal phenotype but retained some basal features. This suggests that IBC derives from a basal cell and progress along a specific oncogenic pathway allowing partial differentiation. This origin may be associated with specific IBC features and aggressiveness even in luminal cases. Although it may contribute, P-cadherin expression may be a sign of but not the reason for this aggressiveness.

## Competing interests

The author(s) declare that they have no competing interests.

## Authors' contributions

ABH and JJ carried out the design of the study and the analysis and interpretation of the data and drafted the manuscript. JJ, ECJ and KM are the pathologists responsible for the diagnosis, dissection of the tumor material and interpretation of the immunohistochemical staining. ISL provided and analyzed clinical data. BE carried out the statistical analysis. PV, SBA, LX and FB were involved in the design. DB participated in the draft of the manuscript and the analysis and interpretation of results. All authors read and approved the final manuscript.

## Abbreviations

CK: Cytokeratin; ER: Estrogen receptor; GTPase: Guanosine triphosphate; IBC: Inflammatory breast cancer; IHC: Immunohistochemistry; MMTV: Mouse mammary tumor virus; NF-kappa B: Nuclear factor; PR: Progesterone receptor; TMA: Tissue microarrays.

## Pre-publication history

The pre-publication history for this paper can be accessed here:


